# Emerging Management Approach for the Adverse Events of Immunotherapy of Cancer

**DOI:** 10.3390/molecules27123798

**Published:** 2022-06-13

**Authors:** Md. Mominur Rahman, Tapan Behl, Md. Rezaul Islam, Md. Noor Alam, Md. Mohaimenul Islam, Ali Albarrati, Mohammed Albratty, Abdulkarim M. Meraya, Simona Gabriela Bungau

**Affiliations:** 1 Department of Pharmacy, Faculty of Allied Health Sciences, Daffodil International University, Dhaka 1207, Bangladesh; mominur.ph@gmail.com (M.M.R.); md.rezaulislam100ds@gmail.com (M.R.I.); sobuj.alam161@gmail.com (M.N.A.); mifoysal1569@gmail.com (M.M.I.); 2 Chitkara College of Pharmacy, Chitkara University, Rajpura 140401, India; 3 Rehabilitation Health Sciences, College of Applied Medical Sciences, King Saud University, Riyadh 12372, Saudi Arabia; albarrati@ksu.edu.sa; 4 Department of Pharmaceutical Chemsitry, College of Pharmacy, Jazan University, Jazan 45142, Saudi Arabia; malbratty@jazanu.edu.sa; 5 Practice Research Unit, Department of Clinical Pharmacy, College of Pharmacy, Jazan University, Jazan 45124, Saudi Arabia; ameraya@jazanu.edu.sa; 6 Department of Pharmacy, Faculty of Medicine and Pharmacy, University of Oradea, 410028 Oradea, Romania; 7 Doctoral School of Biomedical Sciences, University of Oradea, 410073 Oradea, Romania

**Keywords:** immunotherapy, cancer, chemotherapy, immune checkpoint inhibitors, cytokines, interferon-alpha

## Abstract

Immunotherapy, which stimulates the body’s immune system, has received a considerable amount of press in recent years because of its powerful benefits. Cancer immunotherapy has shown long-term results in patients with advanced disease that are not seen with traditional chemotherapy. Immune checkpoint inhibitors, cytokines like interleukin 2 (IL-2) and interferon-alpha (IFN), and the cancer vaccine sipuleucel-T have all been licensed and approved by the FDA for the treatment of various cancers. These immunotherapy treatments boost anticancer responses by stimulating the immune system. As a result, they have the potential to cause serious, even fatal, inflammatory and immune-related side effects in one or more organs. Immune checkpoint inhibitors (ICPIs) and chimeric antigen receptor (CAR) T-cell therapy are two immunotherapy treatments that are increasingly being used to treat cancer. Following their widespread usage in the clinic, a wave of immune-related adverse events (irAEs) impacting virtually every system has raised concerns about their unpredictability and randomness. Despite the fact that the majority of adverse effects are minimal and should be addressed with prudence, the risk of life-threatening complications exists. Although most adverse events are small and should be treated with caution, the risk of life-threatening toxicities should not be underestimated, especially given the subtle and unusual indications that make early detection even more difficult. Treatment for these issues is difficult and necessitates a multidisciplinary approach involving not only oncologists but also other internal medicine doctors to guarantee quick diagnosis and treatment. This study’s purpose is to give a fundamental overview of immunotherapy and cancer-related side effect management strategies.

## 1. Introduction

Immunotherapy is one kind of cancer therapy that revolutionized the treatment of a variety of cancers by boosting the body’s natural defenses against cancer. The importance of tumor-induced immune suppression in tumor progression is overlooked by traditional cancer treatments that aim to halt tumor cells from growing and multiplying [1,2,3]. Antitumor activity in some cancers is promoted by immune checkpoint inhibitors (ICPis) and chimeric antigen receptor (CAR) T-cell treatment through the reduction of immune suppression. These clinical developments could mark a watershed moment in cancer immunotherapy [4,5,6,7]. The most extensively utilized immunotherapy methods–like immunological checkpoint inhibitors–are responsible to reduce a variety of immune checkpoints in active tumor-specific T cells, including cytotoxic T-lymphocyte–associated antigen 4 (CTLA-4) and programmed death 1 (PD-1)/Programmed death-ligand 1 (PD-L1) ICPis. PD-1 is majorly expressed on the T cells of the immune system, whereas PD-L1 is on the cancer cells and antigen-presenting cells. Therefore, the inhibitors that block the interaction of PD-1 and PD-L1 will cause the resurrection of the T-cell mediated anti-tumor immune effect. James P. Allison and Dr. Tasuku Honjo, two cancer immunotherapy experts, were jointly awarded the Nobel Peace Prize in 2018 for their work identifying ways to engage the immune system to target cancer, a breakthrough in generating novel cancer treatments [8]. Antibodies to CTLA-4 and PD-1/PD-L1 have recently been identified for their role in the treatment of a variety of malignancies, including metastatic melanoma [4,9] and nivolumab for non-small cell lung cancer (NSCLC) [10,11]. ICPs have also been shown to improve overall survival (OS) in a variety of cancer subtypes, including renal cell carcinoma (RCC), hepatocellular carcinoma (HCC) [12], and urothelial cancer (UC) [12].

Another promising immunotherapy is chimeric antigen receptor (CAR) T-cell treatment, which uses gene transfer technology to develop a patient’s cytotoxic T cells that consistently make CARs [12]. CAR T-cell treatment targeting CD19 [13] and CD20 has shown great potential in the therapy of malignant tumors of the B-cell type; acute lymphocytic leukemia (ALL) is one example [14,15], as is non-Hodgkin lymphoma (NHL) [14]. Boosting the immune system, despite the promise of clinical findings of cancer immunotherapy in certain malignancies, separates from the regular side effects of standard cancer treatments; immune-related adverse events (irAEs) produce immune-related adverse effects, which are a subset of inflammatory toxicities (irAEs) [16]. The majority of cancer immunotherapy side effects are moderate and manageable with appropriate monitoring and care, however, in rare cases, serious and even life-threatening side effects have been reported. Due to their different techniques, the toxic characteristics of anti–CTLA-4 and anti–PD-1 treatment differ slightly, with anti–CTLA-4 antibodies generating more severe symptoms. The first FDA-approved antibody, apilimumab, has been related to colitis and hypophysitis, whilst nivolumab and pembrolizumab have been linked to pneumonitis and thyroiditis, respectively [15]. Fever, hypotension, and leukopenia are all common CAR-T treatment adverse effects that can be managed.

However, serious issues like cytokine release syndrome (CRS) and severe neurotoxicity (SNT) have been documented [17]. Individuals with less pretreatment immunological markers exhibited higher post-immunotherapy increases in these measures, as well as a higher risk of irAEs, according to a recent study [18]. The use of ICP in patients with symptomatic disease or a history of recent organ transplantation demands a comprehensive examination of potential dangers and advantages [19].

ICPis in conjunction with CTX, targeted treatment, radiation therapy, intratumorally medications, other immunomodulators, or adoptive cell therapy are all being investigated as potential long-term survival improvements [20]. Even though this guideline does not include the management of combination therapy-related toxicities, practitioners need to be conscious of the potential for new toxicity with combination therapy and endeavor to identify the causative agent(s) for optimum care [19,21,22].

Cancer immunotherapy has drastically improved patient survival and quality of life. However, not all tumors are created equal, and there are currently few predictors of response and toxicity. Despite the fast advancements in the area, immuno-oncology is still in its infancy, with many problems and obstacles to overcome. With time, it became clear that the usual tools for evaluating treatment options in the age of chemotherapy and targeted therapies might not be applicable to the new immunotherapies. The Response Evaluation Criteria in Solid Tumors (RECIST) was changed to generate iRECIST, which accounts for the unique patterns of response seen during immunotherapy, such as tumor pseudo progression [23]. Novel methods are essential in the era of cancer immunotherapy, much as TNM staging was critical in directing treatments in the period of chemotherapy. In colon cancer, the Immunoscore has already been shown to offer important prognostic information to TNM staging [24]. As T cells are now widely recognized as major mediators of antitumor success with conventional treatment, the Immunoscore may be an appealing approach for guiding treatment selection in other cancer types as well. However, that option does not rule out the possibility of using additional factors to gain further insight into the peculiarities of each situation. Increasing the efficacy of combination medicines that are already in use in clinical practice is becoming more difficult. In metastatic melanoma, combination CTLA-4 and PD-1 inhibition led to a five-year overall survival rate of more than 50% [25]. In the intention-to-treat population, the same combination has been linked to an overall survival rate of more than 60% at three years in metastatic renal cell carcinoma [26]. Few unique combinations have attained efficacy levels comparable to those new standards of care across the vast landscape of current early-phase clinical studies. Their safety profiles can most definitely be enhanced. In the setting of melanoma, the approved induction and regimen dose of combination icis (ipilimumab 3 mg/kg and nivolumab 1 mg/kg every 3 weeks) is linked to a 59 percent rate of grades 3–4 toxicities [25]. CheckMate 511 demonstrated a considerable improvement in toxicity without losing efficacy [27] while using an alternate dose (ipilimumab 1 mg/kg and nivolumab 3 mg/kg every 3 weeks). Given that iris is sometimes linked to death and considerable long-term morbidity (for example, de novo insulin-dependent diabetes, persistent pituitary dysfunction, or immune-related inflammatory arthropathies), predictors and novel ways to mitigate these side effects are desperately needed. A new treatment for patients that are primary non-responders to ICIS as well as those who develop secondary resistance to these therapies is also urgently needed. Few treatments have been researched beyond ici failure, and doctors frequently rely on previously validated standards of care for each cancer. Early evidence suggests that ICIS exposure may alter the responsiveness to typical therapies administered after progression. After ici failure, for example, extremely high response rates to chemotherapy have been observed on occasion [28,29]. Those findings could be the result of immunotherapy removing the inhibition imposed by tumor cells or other immune cells, followed by cytotoxic chemotherapy-mediated tumor cell death. On the other hand, first-line exposure to ICIS [30] may have a negative impact on progression-free survival and the adverse event profiles associated with targeted therapy (such as braf inhibition in melanoma). To conclude, the future of cancer immunotherapy may rely on combinations of checkpoint inhibitors with tailored cancer vaccines and innovative targeted therapeutics focused on the tumor microenvironment, tumor glycosylation, and the host microbiome, as discussed in this study. Advances in those fields will enable a shift from the current wide “shotgun” approach, in which all cancers within the approved indications are exposed to ICIS, to therapies customized to the characteristics that make each cancer and host a unique coupling [31].

Clinicians must be aware of the symptoms associated with immunotherapy medications, as well as how to monitor and manage them, as their usage in cancer treatment regimens grows. This study’s aim is to investigate current evidence-based breakthroughs in cancer immunotherapy-related symptom management and knowledge.

## 2. Overview of Immune Checkpoint Inhibitor

Tumor cause is associated with the monitoring of the immune system. All malignancies are explained by inherited alterations and germline gene and autonomic genetic changes. The variations in DNA undoubtedly lead to a change in proteins as per the central biological ideology. Neoantigens may play an important role in helping the body make an immune response against cancer cells. Many biologically and biochemically modified novel neoantigens may act as activators, but tumor cells can increase the sensitivity between tumors and cancer stems by changing the binding sites of blocking receptor recognition, allowing them to resist immunologic invasions [32]. The immune system response is regulated through numerous depressant and anti-proliferative elements, as the immunosuppressive method initially implemented by Schreiber et al. in 2002 shows that the immune reaction perhaps consists of three stages of removal, balance, and recovery [33]. Tumor cells perform a key part in the development and implementation of the immunologic indirect indicator [34]. The research for a lung cancer immunotherapeutic rarely comes to an end. At the start of the research, chemotherapy medications focused on malignancy and anti-monoclonal antibody, but they were ineffective [35].

Cancer vaccination Tecemotide is diagnosed with clinical III NSCLC and did not improve treatment outcomes in comparison to the placebo. Currently, the chemotherapy approach has already been transformed from improving the immune system to preventing immunological evacuation. Innate immunity control plays a vital role throughout the natural body in preventing T-cell sophistication and efficiency, maintaining immunogenicity, and eliminating autoimmune conditions. Cancer cells can, therefore, acquire immunological clearance by increased regulation of biochemical mediator production. Consequently, the T-cell mechanism can indeed be reestablished, and immunological stimulation is sustained, as well as the immunosuppressive impact of T-cells boosted by the blockage of immune control molecules against receivers. There are already two immunological control points for treatment that have been approved: CTLA-4/PD-1 immune checkpoints. T cell immunoreceptors with immunoglobulin and ITIM domains (TIGIT) are also investigated (Table 1) [36].

**Figure 1:**
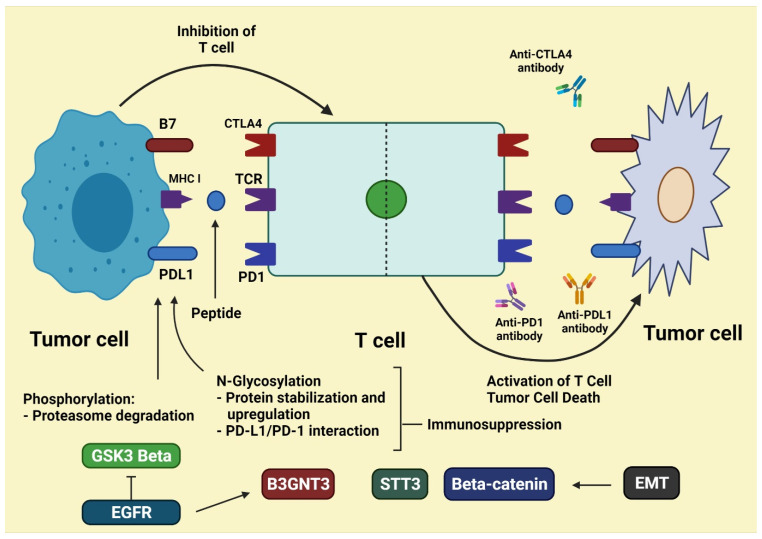
Important targets of immune checkpoint inhibitors approved by the FDA. Degraded proteins are presented on major histocompatibility complex (MHC). Class I proteins expressed on the surface of all the human cells including cancer cells and the MCH class I- peptide complex are recognized by T cell receptors (TCRs). B7 family ligands (CD80 and CD86) can bind to cytotoxic T lymphocyte antigen 4 (CTLA4) expressed on the activated T cells. PD-L1 and PD-L2 expressed on the cell membrane of tumor cells can bind to PD-1 expressed on T cells, which might inhibit T cells through T cell energy and/or apoptosis. Cancer cells can be destroyed by an antibody that attaches to inhibitory receptors on T cells or their ligand on tumor cells. T-cell cytotoxicity is a word that describes T-cell toxicity. T-cell cytotoxicity is initiated and induced as a result. Apilimumab, pembrolizumab, and nivolumab are examples of FDA-approved immune checkpoint inhibitors; atezolizumab, pembrolizumab, and nivolumab, respectively, target CTLA4, PD-1, and PD-L1. Pembrolizumab, nivolumab, and nivolumab/ipilimumab have all been authorized for use in the treatment of colorectal cancer [43].

Tumor cells use co-stimulatory and co-inhibitory signals to prevent being killed by immune cells. CTLA-4 and PD-1 co-inhibitory receptors on T cells, as well as their ligands produced by cancer cells, are the targets of immune checkpoint inhibitors (ICIs) [38,39]. This sparked a surge in melanoma immunotherapy research, with antibodies targeting CTLA-4 and PD-1 proven to be very effective. These antibodies are not effective against advanced melanoma, making treatment difficult for clinicians [22]. When an immune checkpoint inhibitor (ICI) is utilized, T cells are stimulated, and their cytotoxic activity is induced. CD8+ T cells, CD4+ T cells, and macrophages are highly infiltrated in dMMR-MSI-H tumors, and the tumor microenvironment comprises more type I interferons than other CRCs [40,41]. Stage 4 dMMR-MSI-H malignancies make up about 2–4% of all metastatic CRCs, however they have greater levels of PD-1, CTLA4, and PD-L1, making them more susceptible to immune checkpoint drugs [42]. Figure 1 depicts some key targets of immunotherapeutic medicine mechanisms in colon and rectal cancer.

## 3. Atypical Patterns of Responses

RECIST (response evaluation criteria in solid tumor) uses the solid tumor effectiveness criteria to assess the efficacy of chemotherapeutic and fast therapy in the treatment of malignancies. Chemotherapeutic reactions and radiotherapy have two parameters, reaction and progression, however there are many more unusual response patterns [44].

### 3.1. Delayed Response

When commencing an immune response therapy with PD-1/PD-L1, the t cells’ immunologic system must recognize and destroy white blood cells in tumor cells on a daily basis. The result is a therapeutic effect that is distinct from both pharmacologic and immunotherapy: a delayed response. We have noticed three things: (i) tumor cells shrinking in therapy, additional constriction, and possibly a missed diagnosis chance [45]; (ii) the initial phase of immune response therapy did not show significant changes in malignancy, and RECIST was classified as a stable disorder (SD), however after ongoing treatment or therapy, the biopsy started to shrink or vanished; and (iii) the cancer cells were significantly increased or new sores appeared, and RECIST was assessed as the disease progressed. If the tumor is treated with specific antibodies, it may shrink. The average initial period of PD-1/PD-L1 is 2.1 to 2.8 months [46,47]. Treatment methods and cancer treatment are extremely slow; supervisors can be suspicious about the effectiveness of chemotherapy throughout that period. Consequently, the latest indicator must substitute a radiological assessment to know the impact of chemotherapy. However, this impact is so much higher than that of the computed tomography assessment thanks to the changes occurring in circulatory cancer cell DNA (ctDNA). The average opportunity to prove ctDNA impact was 24.5 days, and 72.5 days for X-ray analysis, i.e., 48 days before the radiological analysis [48].

### 3.2. Hyper Progression

In 2016, the European Society of Medical Oncology reviewed 89 instances of NSCLC for the first time, including eight immunotherapeutic cases (8% of which received hyper-progressive chemotherapy (HP)). The HP Definition requires that (i) progress, or a progression duration (TTP) of 2 months, be the first assessment after chemotherapy; (ii) cell proliferation size rise by more than 50%; and (iii) the tumor rate of increase (TGR) increases by two times. The concept of HP must be clarified; Dr. Tourneau described a palm identification when HP is assumed at the 2018 American Society of Clinical Oncology Conference (ASCO): (i) pharmacological evaluation, (ii) scanning performed, and (iii) tumor biopsies. The aim is to prevent insufficient early intervention and move towards yet again another chemotherapy that is possibly involved. It is an issue to conduct the very first assessment after chemotherapy, however, a PSPD evaluation can be assessed close to the end of chemotherapy, and the PSPD can be used as PD to distinguish the associated answers. It has been shown that at 12 weeks of treatment is a crucial time to identify the effectiveness. For RECIST and IrRC, the two-year percentage of starvation as SD, RECIST as PD, and irRC as Non-PD, are measured at 77.6, 37.5, and 17.3 percent, respectively, including both as the non-PD at 12 weeks [49]. To accomplish early diagnosis and aggressive diagnosis of HP, the iRECIST explores the issue of early cancer analysis during studies (e.g., 4–6 weeks rather than 8–12 weeks after treatments begin) [50].

## 4. Immune Checkpoints in Cancer

T cell identification and action versus tumor antigens necessitate dual T cell receptors to bind with antigen peptides delivered inside the environment of major complexes of histocompatibility, as well as costimulatory receptor activation of CD28 on T cells and CD (80/86) on those cell that have antigen-containing cells, or cancer cell (tumor) [51]. Thus, the immunity of anti-tumor may decrease by action of 4[CTLA-4] and PD-1; these are immune check points. This CLTA 4 helps inhibit receptors; it generally works Versus CD-28 for CD 80 or 86. Moreover, they have a high attraction to bind with CD 80 or 86 [51]. T cell energy is signaled by regulation of the CTLA4 pathway rather than T cell stimulation, which occurs when CD28 attaches with CD 80 or 86. In same way, PD-1 attaches with its ligand (PD-L 1 or 2); this happens on cells that contain antigens or tumor cells, and affects the decrease of t cell division and decrease the lifetime of cell. When there is no cancer, a reaction occurs among t cells and antigens of self against non-self [52]. This reaction is controlled by PD-1, CTLA4, and so this suppression influences the opening of auto operative t cells, resulting in irAES and relating to check point inhibition (immune system) [52]. Thus, the treatment of irAEs changes the input from the treatment of cytotoxic-chemotherapy side effects [53].

### 4.1. Immune Checkpoint Inhibitor (ICI) Approved for dMMR-MSI-H Cancers

Immunotherapy has yet to be proven effective in patients with dMMR-MSI-L, who make up the vast majority of metastatic CRC patients. Medication with pembrolizumab had no effect upon dMMR-MSI-L CRC patients [43]. A modest response was seen in one out of every 20 dMMR-MSI-L CRC patients treated with a mixture of anti-PD-1 and anti-CTLA4 antibodies. A mixture of PD-1 inhibitors and other immune checkpoint enhancers may be useful for a few people with dMMR-MSI-L, but different combinations must be researched for the most of CRC patients. Table 2 summarizes some of the most well-known dMMR-MSI-H CRC drug testing employing immunotherapy drugs at various levels of development.

### 4.2. CLTA4 Checkpoint Inhibition and Therapy

**Figure 2:**
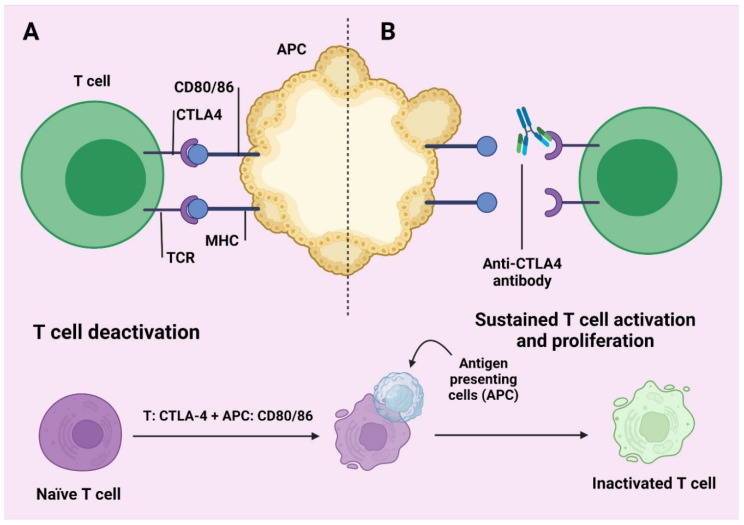
T cell deactivation (**A**) and T cell activation and proliferation (**B**). One (same antigen given through an APC) and signal two: CD-28 interacting with CD 80 or 86, energize the T cell in the lymph node, so that it can help to increase cell numbers and help the activities of T cells [56]. Activities enhance the evolution of the t cytotoxic surface size of the cell, which is related to CLA4. This can combine with CD 80 or 86 rather than 28. These activities are in advance of the checkpoint and are responsible for inactivating the T cell. This middle checkpoint is refused by anti CTLA-4 and the ambit for t cell delegation falls, growing T cell operation [55].

CTLA-4 is still an inhibiting transcription factor that synthesizes the initial phases of the proliferation of T and the very first molecular diagnostic mechanism checkpoint to the destination. CTLA-4 is morphologically similar to CD2, still generated on the immunological cellular membrane, and produced on the cell surface. Regrettably, this is also the case with molecule B7. Furthermore, B7 is closer to CTLA-4 than CD28. CTLA-4 binds B7 to provide an inflammatory response by inhibiting the routes of CD28/B7-1 and B7-2, which suppress T-cell propagation and stimulation [54]. CTLA-4 agonists have the ability to lower CTLA-4 binding to B7 and weaken the B7-1 and B7-2/CTLA-4 suppression systems that allows T cells to perform their anti-tumor functions more effectively. CTLA-4 blockers include ipilimumab and tremeimumab [44]. A 4-monoclonal anti-CTLA inhibitor causes a pathological change mainly in melanoma-based sufferers, but there are several therapeutic NSCLC chemotherapy reports outlining the lymphocytes proliferated inappropriately following inhibition of CTLA-4. This effect can be attributed to severe autoimmune impact and led to widespread proliferation in tissues and organs (Figure 2) [55].

### 4.3. Inhibition of the PD-1 and/or PD-L1 Checkpoints, as Well as Treatment

**Figure 3:**
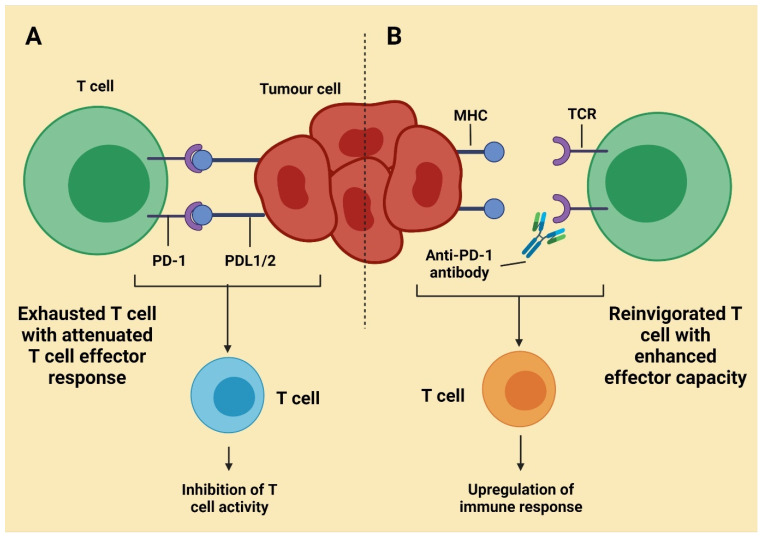
Inhibition of T cell activity (**A**) and upregulation of the immune response (**B**). Activity of CTLA-4 is generally in the tissue of lymph nodes, which are secondary; PD-1 is manifested into T cells, transported from the lymph side, and works on the tumor cell. These cells are peripheral tissue. PD-1 interacts with apoptosis ligand one, found in many tissues, and PD-L2 is is limited for allergen containing cells [58].

In lymphocytes, innate immune cells (NK), macrophages, and B cells, PD-1 are generated. The PD-l1 occurs mainly on malignant cells membranes and in the environment of the tumor. T cells are suppressed when PD-1 attaches to PD-L1. PD-L1 can be inappropriately incorporated in the cancer cells’ mucosa, blocking lymphocyte proliferation, resulting in cell dedifferentiation [57]. Both PD-1 and PD-L1 inhibitions will allow lymphocytes to recover the capacity to comprehend tumor cells and target them, inhibiting their immunological flight. The PD-1 antagonists contain atezolizumab, durvalumab, avelumab, etc., also include nivolumab and pembrolizumab. Latest innovations aside, the cell lung cancer first step and second-string treatments are all attributed to PD-1/PD-L1 Blockers (Figure 3) [44].

The check-point suppression of the PD-1 channel is not simple; FDA provided medicines target both PD-1 and PD-L1. The PDL-1 suppressor supplanter of PDL-2 is effective in cases of effectual clusters of differential 4 (CD4+) cells conciliated in the immune system [8]. The repercussions of this uniqueness are still to be fully appreciated; however, some research shows that significant immune-related side effects can arise [59]. Immune checkpoint inhibitors have several clinical trial outcomes for different types of cancer (Table 2).

**Table 2:** Immune checkpoint inhibitors have shown to be effective in a variety of cancers in clinical trials [38].

Target	Drug	Condition	Treatment Regimen	Treatment in Control Group	Objective Response Rate %	Reference
Programmed cell death protein 1 (PD-1) signaling	Nivolumab (IgG4a)	Melanoma (stage III/IV)	3 mg/kg/2 week	Combination therapy	43.7	[60]
		Renal cell carcinoma (metastatic)	3 mg/kg/2 weeks	10 mg/day Everolimus	25 (4% control)	[61]
		Hodgkin’s lymphoma (relapsed/refractory)	3 mg/kg/2 weeks	n/a	87	[62]
		Squamous-cell carcinoma of the head and neck (recurrent)	3 mg/kg/2 weeks	Single-agent systemic therapy (methotrexate, docetaxel, or cetuximab)	13.3 (5.8% control)	[63]
		Ovarian cancer (platinum-resistant)	1 or 3 mg/kg/2 weeks	n/a	15	[64]
	Pembrolizumab (IgG4a)	Melanoma (stage III/IV)	10 mg/2 weeks or 3 weeks	(vs. ipilimumab)	33.7–32.9	[65]
		Merkel cell carcinoma	2 mg/kg/3 weeks	n/a	56	[66]
		Progressive metastatic colorectal cancer	10 mg/kg/every 2 weeks	n/a	40/0	[67]
	Pidilizumab (IgG1)	B cell lymphoma (after autologous stem cell transfer	1.5 mg/42 days	n/a	51	[68]
		Follicular lymphoma (relapsed)	3 mg/kg/4 weeks (+rituximab)	n/a	66	[69]
T-lymphocyteassociated protein 4 (CTLA-4) signaling	CTLA-4Ipilimumab (IgG1)	Melanoma (stage III/IV)	10 mg/kg plus decarbazine	Decarbazine alone	15.2 (10.3% control)	[70]
			3 mg/kg/3 weeks	(vs. Pembrolizumab)	11.9	[56]
			3 mg/kg/3 weeks	(vs. combination with nivolumab)	19	[60]
	Tremelimumab (IgG2)	Melanoma (stage III/IV)	15 mg/kg/90 days	Chemotherapy (temozolomide or dacarbazin)	10.7 (9.8% control)	[71]
Combination therapy	Nivolumab + Ipilimumab	Melanoma (stage III/IV)	3 mg/kg/2 weeks Nivolumab 3 mg/kg/3 weeks Ipilimumab	(vs. single)	57.6	[60]
		Non-small cell lung cancer	Nivo + Ipi: 1 + 3 or 3 + 1 mg/ml	Nivolumab alone	23/19 (10% control)	[72]

## 5. Immune Related Adverse Event Patterns

Pembrolizumab and nivolumab are authorized drugs for the treatment; both are PD-1 inhibitors. Immunotherapy was reported to have a smaller number of negative effects overall when compared to cytotoxic chemotherapy [73]. In any case, the types of side effects differed significantly between immune check point suppressor therapy and chemotherapy [73,74].

Frailness, drowsiness, vomiting, and loss of motion can occur as side effects of immune treatment and chemo-related therapies, which are related to anemia, infections in the stomach, etc. [73]. The same types of undesired effects can be observed with CLTA 4 suppressor, which suggests that cytotoxic therapies are less bearable than immune check point suppressors; research expressed that CTL-4 suppressor is absent with chemotherapy. Negative effects related to immunotherapy can create an effect on body systems called irAEs [75]; CLTA4 has more value and brutality of irAEs than PD one suppressor [76] in 70–90% of people [77]. Although they have been observed to occur around 3–6 months after starting CTLA4 or PD-L1 antagonist treatment, irAEs frequently emerge in a dose-dependent way as a feature within the year prior or post a person has been subjected to PD-1 antagonists [77]. This seems to become the antagonist among other side effects such as tumors, or like hyperpigmentation in melanoma patients [77].

## 6. Mechanisms Underlying irAEs (Immune Related Adverse Events)

When ICI extended the life expectancy of patients with incurable cancer, there was good feedback alongside some undesirable effects (irAEs) [78]. These effects can create a limitation in the case of providing medication and therapies. IrAEs cause harm to the GIT, respiratory system and cardiac system, and cause hormonal issues, osteoarthritis, dermatitis, etc. [79].

**Figure 4:**
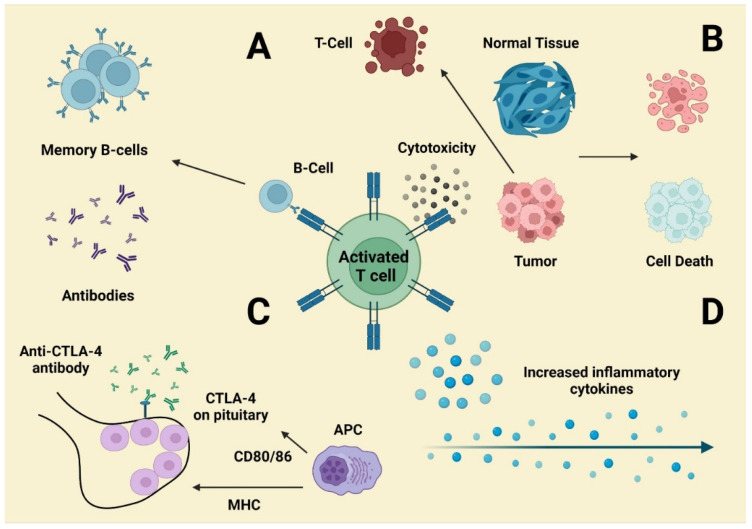
Mechanisms underlying irAEs. (**A**) Tumor-reactive T cells may have an impact on B cell antibody production, resulting in antibody-mediated diseases. (**B**) T cells can infiltrate normal tissues that share an antigen. (**C**) Pituitary dysfunction is likely connected to ADCC produced by ectopic CTLA-4 expression. (**D**) Activation of Th1 and Th17T cells leads to an increase in inflammatory cytokine output, which is especially visible in colitis [82].

Despite cancer immunotherapy’s enormous potential, its therapeutic effects in known indications have yet to be established. The failures and toxicities of cancer immunotherapy are determined by the immunosuppressive extracellular matrix, which collaborates to obstruct innate immunity and immunotherapy efficacy via numerous routes. The tumor microenvironment contains Treg cells, MDSCs, T cells, TAMs, and other inhibitory immunological checkpoints that may play a role in lowering anticancer immune action while limiting autoimmunity. By blocking these signals, ICPs impair immunological integrity, which can result in a range of autoimmune reactions. The impact of ICP therapy in mice models is connected to a greater ratio of Teff to Treg cells. As a result, removing Treg cells from their extracellular environment may have a beneficial effect (Figure 4).

T lymphocytes that have become overly active may injure normal tissues that display the target antigen, putting healthy cells at risk in addition to targeting viral antigens. Regular cells produce neoantigens, cancer antigens, and auto-antigens in reaction to cell disruption caused by cytotoxic T lymphocytes, worsening the injury by ‘targeting’ body cells [80]. Furthermore, activation of Th1 and Th17 T cells after CAR-T cell therapy increases serum cytokine output, like IFN-, IL-17, and IL-6, which typically leads to cytokine release syndrome (CRS).

A relationship between the gut microbiome and ICP-related colitis has been discovered in several studies. On one hand, microbiota components such as Bacteroides spp. and Burkholderiales have been shown to increase Th1 immune activity, which boosts anti-CTLA-4 treatment efficacy. Certain gut microbes, particularly gram-positive microbiota, have, on the other hand, been linked to the beginning of inflammatory diseases. Lactobacillus reuteri probiotics [81], for example, may reduce the number of group 3 innate lymphoid cells in the mucosa, reducing colitis after ICP therapy (ILC3s).

## 7. Main Features of irAEs

### 7.1. Diversity

Most human organs are strongly linked to an overactive immune system because of irAEs (immune-related adverse events), including the skin, endocrine tissue, intestines, liver, kidneys. The CNS (central nervous system) may also be impacted [83]. In irAEs, the most prevalent signs are GIT (gastrointestinal) and dermatological problems [84]. Skin toxicity, which include rash and mucositis, affects more than 30% of patients who are treated with immunotherapy [85]. GIT issues such diarrhea and colitis have also been documented in 30–40% of those who have been given anti-CTLA-4 antibodies [72]. Some of the less prevalent adverse effects, such as endocrine, nephritis, pancreatitis, and neurological diseases, should not be overlooked [58,86,87].

### 7.2. Hysteresis

Dermatological side effects are most common during the first two weeks after starting an immune checkpoint inhibitor medication. Around six weeks after initiating medication, GIT side effects are common [88,89]. Hepatitis can emerge anywhere from 1–49 weeks after the beginning of treatment, with a median of five weeks [90]. Ipilimumab has a 7-week median onset of endocrine toxicity, while nivolumab has a 10-week median onset of endocrine toxicity [91]. Generally, immune-related pneumonitis occurs 8–14 weeks after the commencement of therapy [86].

### 7.3. Unpredictability

As per clinical accounts, irAEs can develop anywhere, at any moment, and span a wide range of symptoms impacting many organs during treatment [76], even leading to death; as a result, they require prompt diagnosis and treatment. Much research has been undertaken, and CD177 and CD80 are two neutrophil activation markers that have been discovered. CEACAM1 has the potential to be a biomarker for GIT toxicity, especially for ICPis toxicity. However, perhaps neither of these indicators will be approved for clinical tests to support diagnosis and effective preventive interventions due to inaccurate pathophysiology [92].

## 8. Diagnosis and Management of irAEs

### 8.1. Dermatological: Rash and Pruritus

The most common side effects include rashes, vitiligo, and pruritus, which are all dermatological reactions; however, additional side effects have also been connected to ICPis (immune checkpoint inhibitors). Rashes and pruritus are now prevalent in individuals on anti-CTLA-4 medicines, which are responsible for more than 40% of those who take ipilimumab. Around 20% of people on PD-1/PD-L1 blockers have experienced this side effect [93]. Bullous pemphigoid [94] skin changes resembling scleroderma [95] and severe cutaneous bad responses (SCARs) [20] are examples of significantly lower commonly diagnosed toxicities. In the first two weeks after starting treatment with an immune checkpoint inhibitor, dermatological side effects are common. However, toxicity might occur at any moment. Vitiligo, as an example, only appears later after many months of ICPis medication [96]. Because dermatological toxicities are common, thorough medical records of immune-related skin problems are important for each immunotherapy patient [93].

To assess the number and kind of skin lesions, a complete medical examination focusing on the skin mucous membrane is required [85], as other etiologies must be ruled out [20]. Whenever a possibly serious diagnosis is being evaluated, several specialized accessory investigations can help to identify the order of seriousness. Patients with eosinophilia and systemic symptoms who have had a drug reaction are given hepatic and renal function testing [93,94]. For autoimmune disorders such as lupus or dermatomyositis, targeted serologic investigations such as the ANA test are required [20]. Whenever rashes become difficult to treat or individuals are identified as having SCARs (severe cutaneous adverse reactions), a skin biopsy is required [93,95].

Topical creams and mild–moderate strength topical corticosteroids are the first line of treatment for a reduced rash with ICPis [20,85]. In the meantime, minimizing skin allergens and excessive sun exposure is critical for preventing damage [20]. Cold compresses and oatmeal showers have been described as effective treatments for pruritic complaints. If the illnesses are affecting one’s life quality, oral antihistamines with medium to high effective topical corticosteroids should be used until the skin diseases subside to category 1 [20]. Although most skin eruptions are mild and most people can maintain cancer treatment [85], there are some rare but serious catastrophic dermatological events: DRESS and Stevens-Johnson syndrome are two examples. In cases like these, ICPis treatment should be stopped once no improvement has been seen as a result of these therapies until a dermatologist can determine if there is a hope of healing [96,97].

### 8.2. Gastrointestinal: Diarrhea and Colitis

GIT illnesses are amongst the most frequent irAEs, yet it is uncertain if immunotherapy causes GIT side effects directly [98]. The most typical causes of GIT toxicity are colitis and diarrhea. Anti-CTLA-4 medication has been seen to increase the incidence of all diarrheas in patients undergoing ICPis up to 30% [99], or 44% in the case of a combined treatment [102]. Patients also feel unusual digestive symptoms like stomach discomfort and vomiting. GIT adverse effects are commonly brief and minor, appearing after around six weeks after starting treatment [8]. However, when nivolumab is combined with ipilimumab, a high grade of diarrhea is recorded in more than 9% of patients [100]. Patients that have ileal rupture are relatively uncommon following immunotherapy for terminal ileitis except colitis, especially when used in tandem [101].

It is difficult to tell the difference between colitis and diarrhea. In addition to blood tests to determine the inflammatory response in colitis, stool samples are sent for etiological testing to rule out infection [102]. A whole-blood mRNA signature, according to some research, can distinguish category 2 or greater colitis or diarrhea from small diarrhea or colitis in the early stages, allowing for appropriate intervention [103]. Symptomatic therapies are suggested when a category 1 GIT issue happens [104] and immunotherapy can also be maintained with adequate dietary adjustments based on observations of dehydration [20]. IV or oral corticosteroids can be used to treat category 2 colitis, commencing at 1 mg/kg/day for category 2 and 2 mg/kg/day for category 3–4 [102]. ICPis therapy must be stopped at the same time if problems cannot be resolved [20]. After 72 h of high-dose IV steroids, infliximab can be utilized in hormone-refractory individuals, and symptoms can occur within 24 h [105]. It is also worth mentioning that if an intestinal perforation occurs, surgical treatment should begin immediately [101].

### 8.3. Hepatotoxic: Hepatitis

Hepatitis, the most prevalent liver toxicity induced by ICPis, has a place in irAEs, although it is less common than GIT illnesses. Hepatitis develops in less than 6% of patients who receive anti-PD-1 antibodies, around 7% of individuals who receive CTLA-4, and more than 30% of patients who receive PD-1/PDL-1 and CTLA-4 blocking combinations [28]. Hepatitis is also responsible for 16% of all fatal immunotherapy reactions [106]. Hepatitis may manifest itself as an asymptomatic elevation in ALT (alanine aminotransferase) and AST (aspartate aminotransferase) readings, as well as a rise in blood bilirubin levels, after 6 to 14 weeks of treatment [90]. Because hepatitis is often asymptomatic, liver activity tests are recommended for all patients prior to their first therapy session, as well as once or twice weekly if AST and ALT values rise in the event of a worsening to category 2 or higher [103]. Biopsies reveal two unique types histologically: zone 3 hepatitis and pan lobular hepatitis [107].

Immunotherapy [110] has been shown to disclose previous subclinical liver issues, as indicated by the research above. In this scenario, a thorough examination is required to rule out viral hepatitis or other infectious diseases, liver metastases, and AIH (autoimmune hepatitis) [104,108,109,113]. Corticosteroids are suggested as a medication for category 2 or higher hepatitis with indications. Despite the possibility of hepatotoxicity, infliximab may not be prescribed for the management of hepatitis [20,90,107].

### 8.4. Endocrine: Hypophysitis and Thyropathy

Immune checkpoint blockers have been linked to a variety of endocrine side effects, varying from typical diagnoses like headache, nausea, and tiredness, to major or life-threatening indications including renal and thyroid crises [91]. Adrenal, thyroid, and pituitary glands seem to be the most implicated components throughout the endocrine system. Thyropathy and hypophysitis are much more prevalent, with rates of 1.8% and 0–9%, respectively. Hypophysitis [110] is typically classified as both a category 3 or 4 toxicity with a dosages pattern [91,110]. Hypophysitis can cause a variety of symptoms (e.g., headaches, libido loss, mild fatigue, and mood swings). Such symptoms are usually caused by swollen glands or hormone deficiency [111,112]. Due to pituitary failure, it is suggested to test thyrotropin, corticotropin, and luteinizing hormone in peripheral blood via essential laboratory procedures [20,112]. 

Such symptoms are clearly developed and controllable with adequate hormone replacement therapy, making continuing medical immunotherapy feasible. Hormones such as estrogen and testosterone can be supplied as necessary to treat category 1 hypophysitis in people who have no contraindications [20,113]. Whenever patients are identified as having category 2 or higher hypophysitis, immunotherapy must be controlled [114].

However, if there are damaging and serious medical signs, adequate emergency steroid administration is required before replacement treatment [20,114]. Hyperthyroidism and primary hypothyroidism are the most frequent forms of thyropathy, with hypothyroidism being the most common. Hypothyroidism usually appears after four weeks of ipilimumab treatment and 10 weeks of nivolumab treatment [91]. As a result, serum TSH and FT4 levels are measured every 4–6 weeks for screening tests and activity follow-up [20]. As a result, professional experience and contact with an endocrinologist are critical in managing all these toxicity issues. Early detection and HRT can help reduce medical symptoms to the greatest extent possible [115]. Patients must be taken to the hospital and given steroids and betablockers as required to ease symptoms and prevent infection [20,91,115].

### 8.5. Respiratory: Pneumonitis

Immune concerned pneumonitis caused by ICPis treatment is a rather unusual side effect. However, in certain people, it is possible that the toxicity will be severe, or even lethal [116]. Pneumonitis is substantially more common in anti-PD-1 patients than in anti-PD-L1 or anti-CTLA-4 patients [117]. The combination of PD-1 and CTLA-4 inhibition, on the other hand, was found to produce more lung damage than any single immune checkpoint blocker, increasing from 5% to 10% in any category, and 2% for categories 3 to 4 [118]. When on ICPs, patients with preliminary respiratory problems like tachypnea, cough, hypoxia, dyspnea, and frosted glass on pulmonary imaging could be suspicious of pneumonitis rthermore; the symptoms listed previously may not be useful in determining a treatment plan [119].

As a result, physicians should exercise caution when diagnosing and treating pneumonitis [119]. Patients with a large suspicion of pneumonitis should first determine the extent of their condition before making a medical decision [120]. Immunotherapy must be stopped until the patient’s condition improves to a category 1 or lower, and prednisone must be given according to protocol for patients suffering from category 2 pneumonitis [19]. If pneumonitis progresses to category 3 or 4, it is also recommended that ICPis must be stopped, and prednisolone and antibiotics prescribed instead [20,121].

### 8.6. CAR-T Induced: CRS and SNT

CRS (cytokine release syndrome) and neurotoxicity are the most known adverse events of CAR T cell treatment, which usually appear 7 and 21 days after treatment, respectively [18,122]. The rate of neurologic side effects ranges between 40% to 44% in teenagers, but 50% in adults [123]. CRS has been recorded in 77% of patients, with 47% developing category 3 to 4 toxicities [19]. Treatment must be dependent on the severity of toxicity in individuals [124], and early measures for relieving pain should be administered [49]. Corticosteroids, also known as helpful for immunocyte reduction, are also essential for the treatment of CAR T-cell therapy’s major adverse effects [18,122,124].

### 8.7. Rheumatic irAE

There were 136 new Rh-irAE cases, 22 of which were multiplex Rh-irAE cases (18.8%). Symmetrical polyarthritis (33.1%), PMR-like symptoms (12.5%), sicca (8.1%), arthralgias/myalgias (11.0%), and other Rh-irAEs were the most common Rh-irAEs (11.0%). In 17.6% of the population, those with numerous Rh-irAE markers, such as symmetrical polyarthritis and myalgias, were more likely to have sicca symptoms. Other Rh-irAEs included a variety of symptoms that were thought to be associated to ICI therapy but could not be classified as a specific inflammatory rheumatic phenotype like Raynaud’s or adhesive capsulitis. With 65.4% of Rh-irAE patients classified as CTCAE grade 1 or 2, one hepatitis, and one myositis, only two patients in this cohort suffered life-threatening episodes. There were no women in that group, nor were there any deaths associated with irAE. Even though these data were only available for 74 patients, further ICI infusions aggravated Rh-irAE symptoms in 37.5% of cases [125].

### 8.8. Non-Rheumatic irAEs

This batch has a large number of other-irAE. There were 94 incidences in 64 patients. Rashes (17%), endocrinopathies (13.8%), colitis (13.8%), and hepatitis (13.8%) were the most common conditions (13.8%). Additionally, pneumonitis (8.5%), ocular involvement (2.1%), hematologic abnormalities (1.1%), and myocarditis (1.1%) were also reported (4.2%) [125].

### 8.9. Tumor Response to irAE Treatment

Overall, 63.2% of this cohort had a complete or partial cure of their cancer before starting irAE, whereas 19.6% (n = 23) had progressed on ICI treatment. Tumor responses improved in 22.2% of cases, decreased in 7.7% of cases, and stayed constant in 61.5% of patients after beginning treatment for an irAE. Tumor responses were higher in 26.6% (n = 12), 29.4% (n = 5), 33.3% (n = 3), and 12.5% (n = 1) of patients with symmetric polyarthritis, PMR, myositis, or biologics, respectively. In 13.3% (n = 6), 0% (n = 1), 11.1% (n = 1), and 12.5% (n = 1) of the patients, tumor responses decreased. After the study, 61.5% (n = 72) of all customers were in full or partial remission, whereas 15.4% (n = 18) experienced tumor progression. Three patients (2.6%) did not respond to the treatment. Three patients (2.6%) did not react to treatment, while four patients (3.4%) required adjuvant therapy. In addition, 18 patients’ (15.4%) cancer conditions were unknown, while two patients died before the follow-up period was finished [85].

## 9. Cancer Immunology and Cancer Immunotherapy Advances

Cancer immunology is entering its Golden Age after years of failures. As a result of recent advancements in cancer immunology, new cancer therapeutic strategies have evolved. Antibodies that disrupt the immunological checkpoint CTLA-4 and PD-1/PD-L1) pathways for the treatment of melanoma were approved by the FDA in 2011 and 2014, respectively. In March 2015, the FDA authorized Nivolumab, an anti-PD-1 antibody, for the treatment of squamous lung cancer. Antibodies addressing PD-1 or PD-L1 are shown to be effective and safe in a variety of malignancies, including non-small cell lung carcinoma (NSCLC), renal cell carcinoma (RCC), bladder cancer, and Hodgkin’s lymphoma. Adoptive cell transfer has becoming more popular and increasingly common in recent years. Alternative T cell-based treatments for several tumor types are currently being explored. Chimeric antigen receptor (CAR) T technology has demonstrated to be beneficial in treating B cell malignancies, and alternative T cell-based treatments for a variety of tumor types are currently being researched. We will go over the most recent developments in cancer immunotherapy and immunology, including innovative drugs currently in clinical trials and possible techniques that have demonstrated promising results in experimental studies [74,126,127].

Cancer is a complex and dynamic tissue that grows and spreads via a variety of strategies including immune evasion [128]. Indeed, the idea of “avoiding immune destruction” as a new cancer hallmark was included in Hanahan and Weinberg’s updated analysis in 2011 [129]. The relationship between immunity and cancer has been extensively investigated in recent decades [130], and immunotherapy has lately emerged as a promising cancer treatment option [131]. Based on Burnet and Thomas’ cancer immunosurveillance theory, it is now commonly accepted that the immune system is capable of identifying tumor antigens spontaneously and of launching a lethal response via the production of specialized anti-tumoral CD8+ T cells [132]. On the other hand, this spontaneous anti-tumor T cell response eventually fails due to two factors: (1) the process of eliminating cancer cells that express antigens recognized by T lymphocytes is known as cancer immunoediting [133,134]; and (2) the activation of immune suppressive pathways by tumor cells and the tumor microenvironment, known as immune checkpoint activation, which inhibits the initial anti-tumoral T cell response [135,136,137,138].

The concept of cancer immunosurveillance has evolved over time into the more recent cancer immunoediting theory. Activation of an innate and adoptive immune response that kills tumor cells (elimination phase), survival of sporadic tumor cells that trigger immunoediting (equilibrium phase), establishment of low-immunogenic tumors, and an immunosuppressive microenvironment (escape phase) are the three phases of cancer immunoediting [139,140,141]. Crosstalk between immune cells, cancer cells, and the microenvironment results in adaptive immune resistance, which is a normal process. The immune system has a dual duty in this mechanism: it protects the host from tumor growth while also assisting tumor advancement. Because T cells are so important in immunosurveillance, early immunotherapies tried to modify T cells to induce endogenous antitumor immunity [135,136]. Blocking immune checkpoint regulators such CTLA-4 and PD-1/PD-L1 pathway in solid tumors [137], as well as customized T cell therapy in acute lymphoblastic leukemia (ALL), cancer immunotherapy, as a monotherapy or in combination with other treatments, has recently ushered in a new age.

## 10. Blockade of Immune Checkpoints in Cancer Patients

Two promising cancer treatments that have recently gained popularity are immune pattern checkpoint inhibitors (ICPIs) and chimeric antigen receptor (CAR) T cell therapy. Following its widespread use in clinics, a slew of immune-related adverse effects, including autoimmune responses, emerged [137,138]. Even if most adverse events are mild and controllable, and even if atypical symptoms make it difficult to recognize them in time, life-threatening toxicities should not be tolerated. This review discusses immunotherapy and the pathways that lead to irAEs. To improve the efficacy of immunotherapy, we are concentrating on early detection methods and the management of a variety of toxicities, as well as improving the efficacy of toxicant-specific screening [120,137,139,140].

## 11. Future Direction

Cancer drug treatments and targeted therapies are expected to be related to an increased irAE prevalence [35]. The most observed combinational therapeutics for developed hepatocellular carcinoma, thyroid problems (25–35%), and arthralgia (18–20%), were accepted by the end of 2019 for the diagnosis of pembrolizumab and avelumab with the inter protease inhibitor axitinib [54]. Furthermore, tofacitinib improves the provision of specific antibodies–therapeutic agents–to tumor cells by modifying immune cells in an animal model [141]. As the toxicity profile of alternate paths of immunotherapeutic management is affected by the growing attention based on inter chemotherapy, it is essential to see if more regionalized therapeutics impact the occurrence and intensity of irAEs sparked. Supply options are equipped to reduce off-tissue impacts intended to in-living regional and cellular uptake, with different therapeutic substances selected depending on patient objectives. There are several structures such as nanomaterials, trusses, hydrophilic, and cell type specimens for biopsy, even though this allows yet more investigation as to whether these systems reduce irAE developments [45].

While doctors’ knowledge of how to handle irAEs has grown as ICI has become more widely used, some obstacles remain. The intricate network of downstream pathways linked with CTLA-4 and PD-1/PD-L1 amplification must be clarified regarding their influence on irAE profiles as well as oncologic therapy results. In addition, irAEs vary in intensity and localization, with some being more specific to the cancer type being treated and the ICI class employed than others [142,143,144,145,146]. Immune surface receptor clustering, for example, appears to predict endocrine irAEs based on ICI class. Thyroid irAEs from ICIs are more common with PD-1/PD-L1 inhibitors than with CTLA-4 inhibitors. Hypophysitis, on the other hand, is more common in CTLA-4s than in PD-1/PD-L1s. Higher rates of PD-1 and CTLA-4 receptor concentrations at the indicated areas explain both these findings [147]. Aside from receptors linked to ICIs, work has been made in developing assays that pinpoint specific laboratory parameters that can predict the development of irAE. Quantitative T cell subpopulation assessments, T/B cell surface receptor concentrations, autoantibody panels, cytokine levels (particularly IL-17), and eosinophilia are some of the tests available. Despite this improvement, larger investigations are required to establish the feasibility and efficacy of these laboratory research before they can be used in clinical settings [148,149,150,151]. Understanding these pathways can help us gain a better understanding of IO-related immune dysregulation from a scientific and clinical standpoint. Furthermore, steroid-sparing based medicines may provide oncologic patients with additional therapy alternatives. This can help patients with steroid contraindications (DM, metabolic syndrome, psychosis, etc.) by reducing long-term side effects, addressing steroid-resistant irAEs, and providing options for patients with steroid-resistant irAEs. This could allow treating clinicians to utilize steroid-sparing medicines earlier in the treatment process, saving high-dose steroids for symptom progression or more serious conditions in the future [152].

In comparison to ICI monotherapy, the frequency and severity of irAEs are projected to rise as the usage of combination regimens increases. The rates of irAEs by the ICI regimen demonstrate this. In addition, compared to monotherapy, combination treatments may cause synergistic irAE activation through more complex processes. The important combination regimens will be examined in the future, considering the enormous number of ongoing IO clinical trials. The key cellular targets being investigated in conjunction with PD-1/PD-L1 medicines as a fraction of all PD-1/PD-L1 combination trials. The current percentage of total IO trials evaluate combination treatments. Furthermore, in IO-based combination therapy with chemotherapy or targeted therapy agents, it may be difficult to tell if a symptom like diarrhea is due to irAEs or a side effect of the non-IO drug(s) in the combination [75]. Patients with pre-existing autoimmune illnesses were commonly excluded from clinical studies that led to ICI treatment approval. There have been nine investigations that have followed these patients with pre-existing immunologic diseases. Polymyalgia rheumatica, myasthenia gravis, rheumatoid arthritis, and psoriasis/psoriatic arthritis were all found to have the highest rates of autoimmune reactivation/flares with ICI therapy (>50% of patients receiving ICIs) [153]. The basic purpose of irAE prevention is to risk stratify patients prior to therapy. Associated laboratory/clinical data are being studied in a variety of ways in order to better identify high-risk individuals and the most common irAEs by malignancy and ICI class [154,155,156]. Pre-ICI therapies, in which steroids were given before the start of the ICI, were found to have little to no effect on the rates of irAEs in studies [157]. Anti-TNF-alpha drugs have shown success in treating uveitis, colitis, and hepatitis in steroid-resistant irAEs [158]. Additional research has found that cyclophosphamide and mycophenolate for pneumonitis, and methotrexate and hydroxychloroquine for arthritis, are effective for steroid-resistant irAEs [159].

While certain irAEs are solely connected with ICI side effects, research has shown that certain irAEs have positive relationships with oncologic outcomes. Vitiligo and other dermatologic irAEs may be positive prognostic indications for melanoma patients [160]. Thyroid cancer, renal cell carcinoma, and other malignancies irAEs [161,162,163] showed similar relationships with efficacy. In patients with metastatic renal cell carcinoma, side effects linked with non-IO treatments, such as Sunitinib, have comparable relationships and effects on baseline thyroid function [164]. 

**Figure 5:**
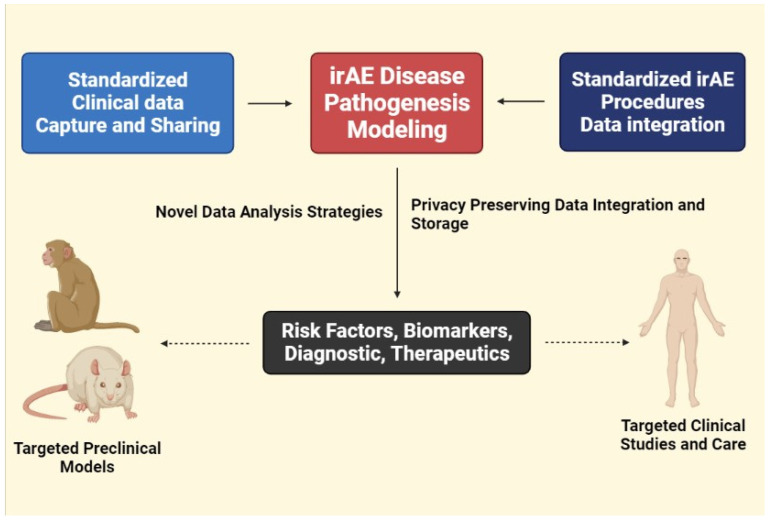
Prediction of new drug development through irAE disease pathogenesis modeling.

Because of the influence on cancer treatment, these irAEs may require more investigation to enable for appropriate ICI continuing. Certain aggressive cancers with complicated connections with the endocrine, humoral, and cellular-based immune systems may require oncologic-specific algorithms. Mixed results have emerged from studies assessing the effects of IO agents on certain high-grade neuroendocrine (HG-NEN) tumors, raising issues about the predictive/prognostic usefulness of PD-1/PD-L1 expression alone for IO deployment and side-effect management [165]. While some NENs, such as melanoma and non-small cell lung cancer (NSCLC), have shown promising outcomes in terms of PD-1/PD-L1 expression and ICIs, some malignancies, such as Merkel cell carcinoma (MCC), require further clinical trial data to better advise IO management [166]. Current debates in the irAE literature revolve around how irAEs are reported, documented, and hence managed. There is significant variability between providers and institutions for irAE reporting outside of endocrine irAEs, which have specified laboratory cutoffs and restricted alternative diagnoses [167]. As a result, uniformity of terminology, documentation, and diagnostic parameters within the irAE research field is critical [167]. Clinicians must consider the costs and benefits of these new IO treatments when dealing with IrAEs. IrAEs are complicated, with both positive and negative relationships with oncologic outcomes. IrAE management algorithms, documentation patterns, and pre-ICI screenings appear to be at the forefront of irAE research right now, and they have the potential to transform how we manage these side effects (Figure 5).

## 12. Conclusions

Cancer immunotherapy offers hope to people with cancer, particularly those with hematologic malignancies, metastatic melanoma, and non-small cell lung cancer (NSCLC). Most immune-related adverse events (irAEs), such as rashes, are modest and treatable with symptomatic and supportive therapies. However, because of the mild and unique symptoms that make early diagnosis difficult, the incidence of unexpected or even life-threatening toxicities should not be underestimated. To aid self-monitoring, patients should be aware of any potential precursory symptoms that may have occurred throughout various stages of treatment prior to starting immunotherapy. For a better prognosis and to avoid toxicity, more scientific and clinical study is required. Standardized diagnosis and management necessitate interdisciplinary collaboration, and more perspectives from many sectors of medicine must be shared to achieve this goal. Additional perspectives from diverse sectors of medicine must be exchanged to achieve the equilibrium.

## Figures and Tables

**Table 1:** Checkpoint inhibitors for cancer immunotherapy have been approved by the FDA [36].

Medications	Molecular Target	Indication	FDA Granted Year [37]
Pembrolizumab	PD-1	1. Melanoma	2014
		2. NSCLC	2015
		3. Hodgkin lymphoma	2017
		4. Urothelial carcinoma	2017
Nivolumab	PD-1	1. Melanoma	2013
		2. NSCLC	2014
		3. Renal cell carcinoma	2015
		4. Hodgkin lymphoma	2016
Durvalumab	PD-L1	Urothelial carcinoma	2017
Ipilimumab	CTLA-4	1.Melanoma	2011
		2.Melanoma in combination with nivolumab	2014
Avelumab	PD-L1	1.Merkel cell carcinoma	2017
		2.Urothelial carcinoma	2017
Atezolizumab	PD-L1	1.Urothelial carcinoma	2016
		2.NSCLC	2016

## Data Availability

Not applicable.
